# Indoor Visual Positioning Aided by CNN-Based Image Retrieval: Training-Free, 3D Modeling-Free

**DOI:** 10.3390/s18082692

**Published:** 2018-08-16

**Authors:** Yujin Chen, Ruizhi Chen, Mengyun Liu, Aoran Xiao, Dewen Wu, Shuheng Zhao

**Affiliations:** 1 State Key Laboratory of Information Engineering in Surveying, Mapping and Remote Sensing (LIESMARS), Wuhan University, Wuhan 430079, China; yujin.chen@whu.edu.cn (Y.C.); amylmy@whu.edu.cn (M.L.); xiaoaoran@whu.edu.cn (A.X.); wudewen@whu.edu.cn (D.W.); photonmango@foxmail.com (S.Z.); 2 Collaborative Innovation Center of Geospatial Technology (INNOGST), Wuhan 430079, China

**Keywords:** indoor positioning, image geo-localization, image retrieval, CNN features, pose estimation

## Abstract

Indoor localization is one of the fundamentals of location-based services (LBS) such as seamless indoor and outdoor navigation, location-based precision marketing, spatial cognition of robotics, etc. Visual features take up a dominant part of the information that helps human and robotics understand the environment, and many visual localization systems have been proposed. However, the problem of indoor visual localization has not been well settled due to the tough trade-off of accuracy and cost. To better address this problem, a localization method based on image retrieval is proposed in this paper, which mainly consists of two parts. The first one is CNN-based image retrieval phase, CNN features extracted by pre-trained deep convolutional neural networks (DCNNs) from images are utilized to compare the similarity, and the output of this part are the matched images of the target image. The second one is pose estimation phase that computes accurate localization result. Owing to the robust CNN feature extractor, our scheme is applicable to complex indoor environments and easily transplanted to outdoor environments. The pose estimation scheme was inspired by monocular visual odometer, therefore, only RGB images and poses of reference images are needed for accurate image geo-localization. Furthermore, our method attempts to use lightweight datum to present the scene. To evaluate the performance, experiments are conducted, and the result demonstrates that our scheme can efficiently result in high location accuracy as well as orientation estimation. Currently the positioning accuracy and usability enhanced compared with similar solutions. Furthermore, our idea has a good application foreground, because the algorithms of data acquisition and pose estimation are compatible with the current state of data expansion.

## 1. Introduction

The increasing demand of location-based services (LBS) in recent years inspires the desire for accurate position information. The most common way for positioning with cell phone and other mobile platforms is GNSS (Global Navigation Satellite System). However, in most of the time, GNSS is only available for the outdoor environment. When it comes to indoor environment, GNSS signals are mostly blocked by obstacles. In recent years, a number of alternative technologies have been proposed for indoor positioning. Most of indoor positioning methods are focused on fingerprinting-based localization algorithms which are infrastructure-free [1,2,3]. In these methods, Wi-Fi received signal strengths (RSS) or magnetic field strengths (MFS) are collected and will be compared with data in a fingerprinting database during positioning period. This fingerprinting-based system is easy to establish and can achieve fine localization performance in the short term. Nevertheless, signal patterns will change over time due to the environment changes, which makes it hard to maintain the positioning performance. Additionally, construction of fingerprint database is time-consuming and labor-intensive. To overcome the defects of this scheme, many alternatives have been proposed, including Optical [4,5], RFID (Radio Frequency Identification) [6], Bluetooth Beacons [7], ZigBee [8,9], Pseudo Satellite [10,11], etc. Whereas the accuracy is not enough in intricate indoor environment, and these solutions may need artificial setting and additional infrastructures which may bring unbearable costs.

There are also some previous attempts at indoor visual positioning. **Recognition-based** image geo-localization methods are quite similar with the problem of image classification in computer vision, in which global or region features are used for image matching [12,13,14,15]. In image classification issue, similar images are labeled as the same category. Regarding the visual localization problem, relative images are identified as sharing similar geo-location information. As for recognition-based method, location of the target image is estimated by retrieving related images or scene classification [16,17,18,19]. Recognition-based methods apply an image retrieval strategy or a scene classification strategy at first, subsequently the location of the query image is estimated based on the localization information of the associate retrieved images or the classification labels. However, the mentioned methods above generally provide a rather coarse estimation of location, which hardly satisfies the need of accurate LBS. **Geometric matching-based** methods represent the scenes by geo-referenced 3D models, and then, estimate the pose of query image by directly matching 2D image features to 3D models or by matching 3D image features to 3D models when depth information is available. These approaches typically come with estimation of 6 degrees of freedom (DoF) camera parameters. However, geometric matching-based methods still have many challenges, which can be concluded as follows: (1) Superior difficulty in constructing high fidelity RGB-D scene models as well as employing 2D-to-3D matching for textured 3D models scheme; and (2) as for non-RGB point-only models scheme, the problem of geometric alignment between the query images and 3D point models can be hard to settle.

To overcome the limitations of recognition-based methods and geometric matching-based methods, a combination of these two strategies has been proposed in the devised scheme. In this paper, we demonstrate an image-based indoor localization scheme which is capable of not only achieving sub-meter level positioning accuracy but also determining orientations. At the same time, the proposed scheme merely uses RGB images in the course of the online localization period and a server is used to host the image database for the computing operation.

The main contributions of this paper can be concluded as follows:

(1) Inspired by the visual spatial cognition ability of human, an image-based visual positioning scheme is proposed. The target image is matched with database images to get the most similar image for localization computing.
(2) Our visual localization algorithm is 3D-modeling-free. Compared with visual localization methods that combine with image retrieval and image pose estimation from regional 3D reconstruction, regional 3D modeling is unrequired in our scheme since we recover camera pose from two sets of 2D-to-2D matches.
(3) Our spatial model is training-free for different scenarios. Owing to pre-trained deep learning models are stable and can be used as powerful feature extractors, we apply deep convolutional neural network (DCNN) pre-trained on ImageNet to extract features to represent images, thus we need not train a unique model for a specific scene.
(4) For localization purpose, we use a lighter model to represent the scene. CNN features extracted from images of database can represent the scene in image retrieval phase. Compared with CNN learning-based visual localization methods that require a large number of images during model training, much fewer images are required to represent the same scene in our scheme.

The paper proceeds as follows: Section 2 provides a brief overview of related work. The system architecture and methods are described in detail in Section 3. Experiments and performance evaluations are presented in Section 4. Section 5 and Section 6 are discussion and suggestions for future work.

## 2. Related Work

The work presented in this paper relates to many fields, such as visual localization, image retrieval, and visual pose estimation.

At present, visual localization systems can be roughly divided into three categories. 

*Structure-based localization methods* are the most common visual localization methods that utilize local features to estimate 2D-to-3D matches between features in a query image and points in 3D models, or employ 3D-to-3D matches between RGB-D images and 3D models. Then camera pose will be estimated from the correspondence. Similarly, Torsten et al. [20] compared 2D image-based localization with 3D structure-based localization, and they drew a conclusion that purely 2D-based methods achieve the lowest localization and 3D-based methods offer more precise pose estimation with more complex model construction and maintenance. They proposed a combination of 2D-based methods with local structure-from-motion (SfM) reconstruction which has both a simple database construction procedure and accurate pose estimation. However, the drawback of their method is significantly longer run-time during the location process.

*Image-based localization methods* were pushed by massive repositories of public geo-labeled images. These methods employ an image retrieval-based strategy [16,17,18,19], which match the query image with images from the database. Afterward the location of the query image is computed based on the pose information of the retrieved reference images [21,22,23]. Owing to the prosperity of social network and street view photos, quantity of images with geo-tags has emerged which can be used for reference to these data-driven image-based localization methods. *Image retrieval* is a visual search task that searches and retrieves images from a large database of digital images, which is commonly used in many image-based localization methods. Conventional methods retrieve images based on local descriptor matching and reorder with elaborate spatial verification [24,25,26]. Content based image retrieval search for images relies on visual content such as edges, colors, textures, and shape [27]. Recent works leverage deep convolution neural networks for image retrieval, the majority of them use a pre-trained network as local feature extractor. Moreover, some work even can address the problem of geometric invariance of CNN features [28,29], and to accurately represent images of different sizes and aspects ratios [30,31].

*Learning-based localization methods* emerged in the past few years, which benefited from the dramatic progress made in a variety of computer vision tasks. By training models from given images with pose information, scenes can be represented by these learned models. These learning-based localization methods either predict matches for pose estimation [32,33,34,35] or directly regress the camera pose such as PoseNet [36], PoseNet2 [37], and VlocNet [38]. PoseNet was the first approach to use DCNNs to solve the metric localization problem, and then Bayesian CNN implementation was utilized to address the pose uncertainty [39]. After that, architectures such as long-short term memory (LSTM) [40,41,42] and symmetric encoder-decoder [43] were utilized to facilitate the performance of DCNNs. 

Moreover, many localization methods [44,45,46,47] adopt a from-rough-to-precise idea. For example [44], to utilized scene recognition to locate in scene-level area, and then employed a multi-sensor fusion approach to give a specific location. Similarly, the purely visual-based methods have also been proposed by researchers. Reference [45] casts the localization as an alignment problem of the edges of the query image to a 3D model consisting of line segments. In Reference [46], recognition-based periods are utilized to give coarse localization and then matching can be employed in rather small region. Whereas, in their work, the accuracy and robustness are not sufficient for pervasive use, for the reason that their SIFT-based images retrieval is not stable for the complexity and diversity of indoor environments. To solve this problem, the proposed method adopts a robust CNN-based images retrieval scheme which can fully satisfy the requirement of image retrieval, which is efficient for indoor scenes. Moreover, 3D model is unnecessary in our strategy.

Compared with previous schemes, this paper combines image retrieval-based strategy with feature-based pose estimation period. During the image retrieval period, we utilize a network pre-trained on ImageNet as feature extractor. CNNs learn suitable feature representations for localization in indoor environments, and experiment shows that the performance of this strategy is sufficient to retrieve spatial adjacent images. Pose of the target image is estimated based on a selected geo-tagged image, this algorithm was inspired by similar procedure in monocular visual odometer which uses the images of nearby frames as well as the estimated pose of the first frame. Due to the procession of 3D modeling is complicated, we utilize a strategy that represents local scenario by two contiguous images and succeeding computed the query image’s pose from one of the reference images. However, the performance of pose estimation is highly related to the similarity between the query images and the reference images. In other words, well-behaved image retrieval paves the way of valid precise pose estimation.

## 3. System Overview and Methods

In this section, firstly, we describe the proposed method at a high level. Then, key modules and important algorithms are described in detail, including data preparation, CNN-based image retrieval and pose estimation.

### 3.1. System Architecture

We demonstrate a single RGB image based localization system which is not only capable of reaching sub-meter localization accuracy but also estimating orientation. 

**Figure 1:**
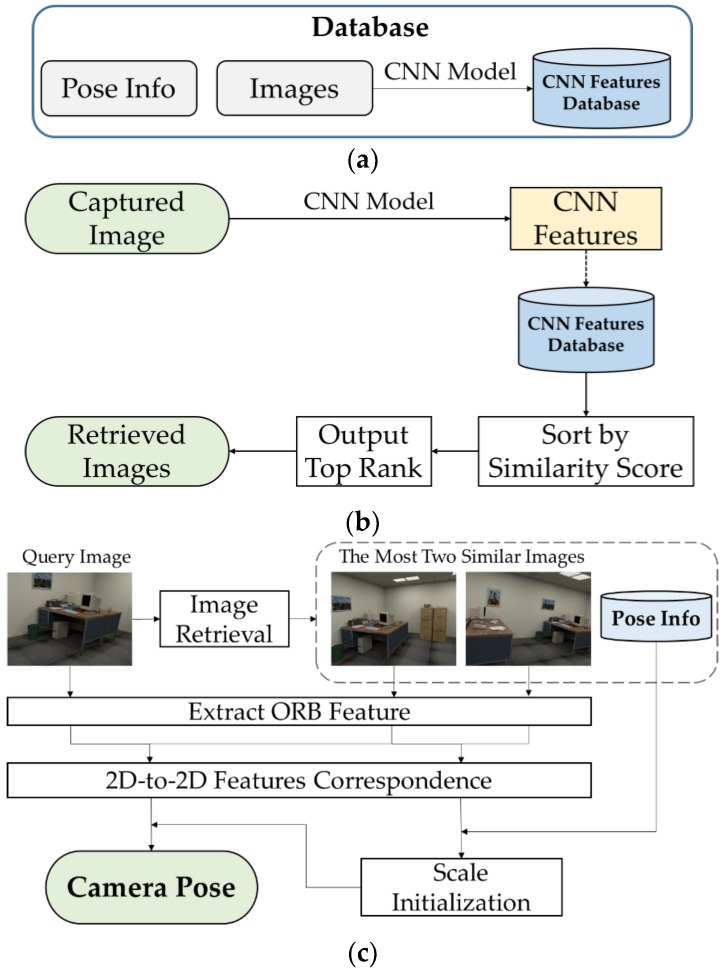
Overview of our visual indoor positioning method. The process is composed of (**a**) database construction; (**b**) image retrieval; and (**c**) pose estimation stages.

The proposed system consists of three components, as shown in Figure 1:

(1) Data preparation, shown in Figure 1a: We collected RGB images from target scenarios, then extracted CNN features from all RGB images through pre-trained CNN models. All of the work was done in offline period.
(2) Image retrieval, shown in Figure 1b: We loaded all of the CNN features of images in database, and ranked them according to their similarity from the CNN features extracted from captured image, and then output a set of images with top similarity.
  Pose estimation, shown in Figure 1c: We carried out image retrieval to the query image and got two of the most similar images as well as their poses. Then, feature points were extracted from the query image and retrieved images. We employed 2D-to-2D correspondence to feature points extracted from two retrieved images to compute the scale in monocular vision setting, and then applied the same procedure to feature points from the query image, and the matching image to compute the pose of the query image.

### 3.2. Data Preparation

In this part, structure of the database is described. The input of the proposed system is an RGB image which is captured either by a cellphone camera or other mobile platforms. In the database, the absolute 3D spatial coordinates (*x*, *y*, *z*) and quaternion (*qx*, *qy*, *qz*, *qw*) of all images are known with respect to a given local coordinate system. In addition, CNN features of each image are also included in image database.

Each image can locally represent the scene it belongs to, and image set contains the information of the scene. In the proposed method, two of the most similar images are applied to compute the scale of monocular vision during pose estimation period, therefore, adjacent images should have enough common area for feature matching. The more well-selected images to represent the scene, the better performance of the retrieval and pose estimation result would be. Besides, too many images result in increasing of the cost of data acquisition and computing time. We design the image set as follows. 

As shown in Table 1, the database ***S*** of this experiment contains n different scenes as $S=\left\{S_{1},\;S_{2},\;\ldots ,\;S_{n}\right\}$. For each scene $S_{i}$, we need to get a set of images $I=\left\{I_{ij}\right\}$ with associated pose information $P=\left\{P_{ij}\right\}$, and their respective CNN features $C=\left\{C_{ij}\right\}$ to create a global representation of this scene, where $P_{ij}=\left\{x_{ij},\;y_{ij},\;z_{ij},\;qx_{ij},\;qy_{ij},\;qz_{ij},\;qw_{ij}\right\}$ is the position and pose data of image $I_{ij}$. 

### 3.3. CNN-Based Image Retrieval

In this section, fundamentals of a deep convolutional neural network are described, as well as a pre-trained CNN model for deep feature extraction in following experiment.

#### 3.3.1. Deep Convolutional Neural Networks (DCNNs)

**Figure 2:**
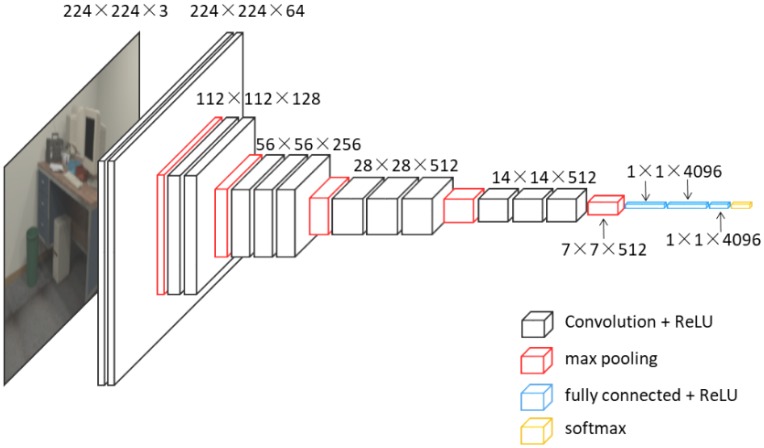
Architecture of VGG16.

As illustrated in Figure 2, the configuration of CNNs used in our proposed scheme is similar to VGG16 which achieved great performance in the large-scale image recognition tasks such as ILSVRC classification and localization. VGG-Nets apply the same principles as normal CNNs, and the key characteristic of this kind of method is increasing depth using an architecture with very small (3 × 3) convolution filters. [48] proposed six kinds of VGG-Nets, number of their layers varied from 16 to 24. In our proposed scheme, we use a 16-layer VGG-Net named VGG16, this network consists of thirteen convolutional layers (block1_conv1, block1_conv2, block2_conv1, block2_conv2, block3_conv1, block3_conv2, block3_conv3, block4_conv1, block4_conv2, block4_conv3, block5_conv1, block5_conv2, block5_conv3), five max pooling layers (block1_pool, block2_pool, block3_pool, block4_pool, block5_pool), three fully connected layers (fc1, fc2, fc3) and a soft-max layer.

It is hard to train a valid DCNN model only by data we collected since deep learning needs a mass of training data. In the proposed scheme, we use CNN for image feature extraction and apply the extracted features in a retrieval task, and then get the most similar images related to the query image. In view of the representation power of CNNs, pre-trained networks based on ImageNet can be used in our feature extraction period. 

#### 3.3.2. Deep Features Extracted by CNNs

As shown in Figure 1a,b, both the query images and images in database are processed by CNN model. From previous work [14], we know that deeper layers represent higher level of sematic information from the visualization of feature maps. In our experiment, deep features extracted from CNN can better represent the image, therefore competitive accuracy of image retrieval can be achieved.

**Figure 3:**
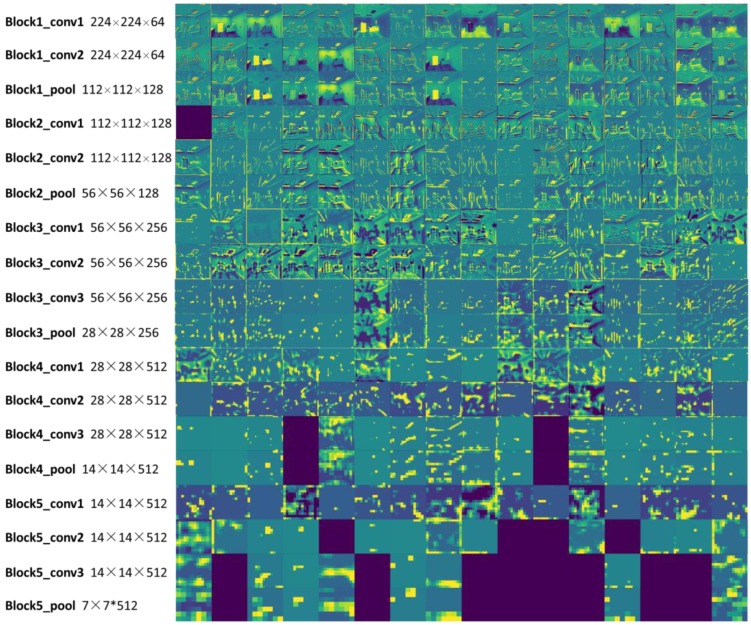
Convolution layers visualization. The first 16 matrices of each layer were visualized, and empty matrices corresponding to dropped out part in CNN. In order to better visualize features in layers, a viridis color map was employed, so layer maps looked greenish.

Convolution layers (including responding ReLU and max pooling) are used to extract features from input images, and these features are robust to scale and translation. Subsequently image features are aggregated into a compact feature vector of fixed length. As is shown in Figure 3, we visualize the first 16 matrices of each layer. We scale layer maps to the same size when visualizing them, but their sizes as well as depths are different among layers as labeled at the left of the figure.

#### 3.3.3. Image Retrieval Using Deep Features

**Figure 4:**
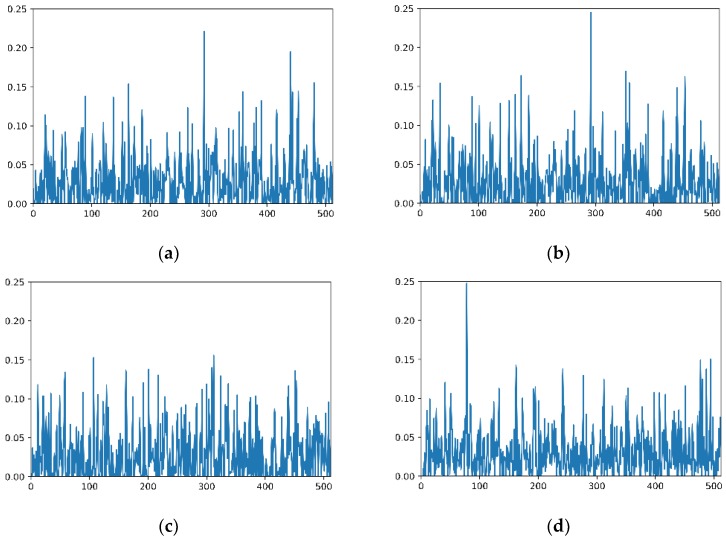
Image feature vectors visualization. (**a**) A shows the vector (512 dimensions) of the query image; (**b**) shows the vector of retrieved image with a top score; (**c**) shows the vector of an unrelated image in the same scene; and (**d**) shows the vector of an image from a different scene.

Image features are aggregated into a vector of fixed length after feature extraction period. If we apply the same CNN model to extract features to the same size images, we will get the same fixed length of feature vectors, as shown in Figure 4. When comparing two images, we calculate the distance between image feature vector of retrieved image $(vector_{i}$) and vector of the query image $\left(vector_{q}\right)$, where i∈[1,n] and n refers to the number of images in database. For a query image, we apply Equation (3) to calculate its scores with every image in database. Then, the images in the database are ranked in descending order of scores. At the end of this period, we output certain number of the most similar images as retrieved images related to the query image.

(1) $$vector_{i}=\left[vector_{i}^{0},\;vector_{i}^{1},\ldots ,vector_{i}^{512}\right],$$

(2) $$vector_{q}=\left[vector_{q}^{0},\;vector_{q}^{1},\ldots ,vector_{q}^{512}\right],$$

(3) $${\mathrm{score}}_{i}=vector_{i}*vector_{q}{}^{T},$$

### 3.4. Pose Estimation

As shown in Figure 1c, the last step of our visual indoor localization process is pose recovery of the query image. In image retrieval period, we get two of the most similar geo-tagged images related to the query image. In this period, the method for pose estimation mainly consists of the following four steps: Firstly, key-points and descriptors are extracted to mathematically express these three images. Secondly, the transformation from 2D-to-2D matches between images is calculated. Thirdly, both the transformation and the pose of the two retrieved images are utilized to compute the scale of monocular vision. Finally, pose of the query images is computed by applying the monocular vision scale and the transformation between the query image and the most similar image. It should be noted that, target images and images in database may captured by different cameras, and the difference in intrinsic parameters may affect the localization performance. Therefore, camera calibration is required beforehand in order to achieve more accurate positioning result. Furthermore, in consideration of top 2 retrieved images may fail to estimate the pose of the target image, all images in database are re-ranked in retrieval period. If the transformation from 2D-to-2D matches between images is calculated failed, we use the next ranked retrieved image for transformation computation, until pose of the target image could be figured out.

#### 3.4.1. Feature Detection and Matching

The proposed system aims at recovering pose of the query image based on nearby image. However, the image is a matrix of brightness and color, and it is hard to compute the transformation between images by whole matrix. The most common approach to this issue is searching for salient key-points that can be used to match well in other images. 

There are many point-feature detectors, such as corner detector (e.g., Moravec [49], FAST [50]) and blob detectors (SIFT [51], SURF [52], CENSURE [53]), their pros and cons can be found in Reference [54]. SIFT fully considers the illumination, scale, rotation and other changes in the image transformation, and achieves great performance in many positioning applications such as in Reference [46], however it can result in large computational cost. For the proposed system which is designed for real-time positioning and LBS, SIFT cannot satisfy the requirements. The time of extracting the same number of features from the same image by SIFT, SURF and ORB [55] is compared in Reference [55]. In that work, when extracting roughly 1000 features, SIFT takes 5228.7 ms, SURF takes 217.3 ms, while ORB only takes 15.3 ms. Accordingly, we adopt ORB (Oriented FAST and Rotated BRIEF) as our detector of point features.

**Figure 5:**
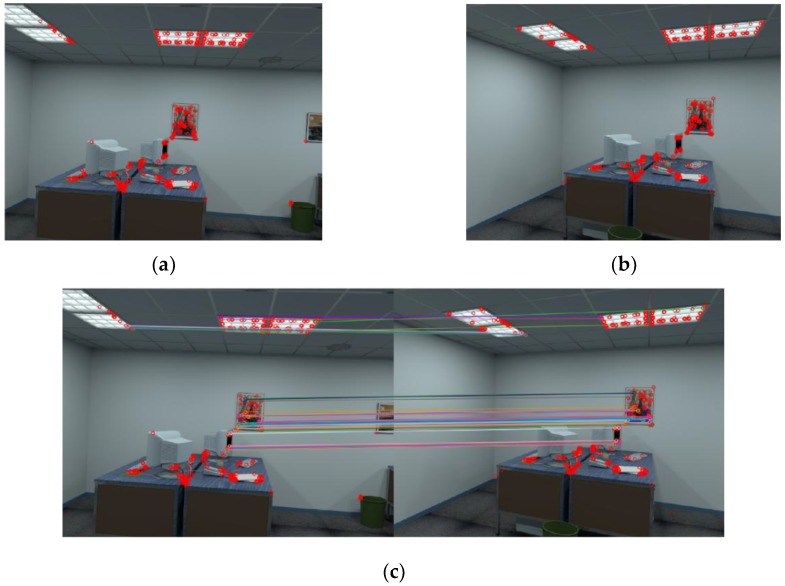
Results of feature detection and matching. (**a**,**b**) show key-points detected in a pair of images; and (**c**) shows the first 50 matches.

After extraction of ORB features from a pair of images, we use Hamming distance as distance measurement to match features. The results of feature detection and matching from two images are shown in Figure 5.

#### 3.4.2. Motion from Image Feature Correspondences

In our proposed system, only RGB images are captured by cameras, therefore, feature correspondence is in two dimensions. This section explains the method of computing the transformation $T_{K}$ between two images $I_{K-1},\;I_{K}$ from two sets of corresponding features $f_{K-1},f_{K}$.

For calibrated cameras, the geometric relationship between two images $I_{K-1}$ and $I_{K}$ can be described by essential matrix *E*. The rotation and translation can directly be extracted from *E* which can be computed from 2D-to-2D feature correspondence. *E* contains an unknown scale factor of the transformation parameters in the following form:

(4) $$E=\hat{t}R,$$

where $t={\left[t_{x},t_{y},t_{z}\right]}^{T}$ and

(5) $$\hat{t}=\left[\begin{array}{ccc} 0 & -t_{z} & t_{y}\\ t_{z} & 0 & -t_{x}\\ -t_{y} & t_{x} & 0 \end{array}\right],$$

*E* can be computed from 2D-to-2D feature correspondence by using the epipolar constraint. Reference [56] illustrated a minimal case solution, which involves the correspondence of five pairs of points’, and Reference [57] proposed an efficient implementation of this five-point-algorithm. In References [58,59], an eight-point-algorithm is created for the n≥8 noncoplanar points, which is summarized below. For one pair of points with normalized coordinates $x_{1}={\left[u_{1},v_{1},1\right]}^{T}$ and $x_{2}={\left[u_{2},v_{2},1\right]}^{T}$, according to epipolar constraint as Equation (6), where $E={\left[e_{1},e_{2},e_{3},e_{4},e_{5},e_{6},e_{7},e_{8},e_{9}\right]}^{T}$.

(6) $$\left[u_{1}u_{2},u_{1}v_{2},u_{1},v_{1}u_{2},v_{1}v_{2},v_{1},u_{2},v_{2},1\right]E=0,$$

Stacking the constraints from eight points gives the linear equation system as Equation (7), and the parameters of *E* can be computed by solving this system.

(7) $$\left(\begin{array}{ccccccccc} u_{1}^{1}u_{2}^{1} & u_{1}^{1}v_{2}^{1} & u_{1}^{1} & v_{1}^{1}u_{2}^{1} & v_{1}^{1}v_{2}^{1} & v_{1}^{1} & u_{2}^{1} & v_{2}^{1} & 1\\ u_{1}^{2}v_{2}^{2} & u_{1}^{2}v_{2}^{2} & u_{1}^{2} & v_{1}^{2}u_{2}^{2} & v_{1}^{2}v_{2}^{2} & v_{1}^{2} & u_{2}^{2} & v_{2}^{2} & 1\\ & \vdots & & \vdots & & & \vdots & & \\ u_{1}^{8}v_{2}^{8} & u_{1}^{8}v_{2}^{8} & u_{1}^{8} & v_{1}^{8}u_{2}^{8} & v_{1}^{8}v_{2}^{8} & v_{1}^{8} & u_{2}^{8} & v_{2}^{8} & 1 \end{array}\right)\left(\begin{array}{c} \begin{array}{c} e_{1}\\ \begin{array}{c} e_{2}\\ e_{3}\\ e_{4} \end{array}\\ e_{5} \end{array}\\ e_{6}\\ \begin{array}{c} e_{7}\\ e_{8}\\ e_{9} \end{array} \end{array}\right)=0,$$

The rotation and translation can be extracted from *E* using singular value decomposition (SVD). A valid essential matrix after SVD is $E=USV^{T}$. Generally, four different solutions for *R*, *t* for one *E*; however, by triangulation of a single point, the correct *R*, *t* can be determined. The four solutions are:

(8) $$R=U\left(\pm W^{T}\right)V^{T},$$

(9) $$\hat{t}=U\left(\pm W^{T}\right)SU^{T},$$

where

(10) $$W^{T}=\left[\begin{array}{ccc} 0 & \pm 1 & 0\\ \mp 1 & 0 & 0\\ 0 & 0 & 1 \end{array}\right],$$

The transformation can be described by transform matrix *T* in the following form:

(11) $$T=\left[\begin{array}{cc} R & t\\ 0 & 1 \end{array}\right],$$

#### 3.4.3. Scale Determination

The main property of 2D-to-2D motion estimation is the epipolar constraint, which is based on the constraint of zero equation. Therefore, the equivalence is valid when essential matrix multiplies a multiplicative scalar. In other words, the essential matrix lacks scale to merely correspond to real scenario. This section explains the method to determine the scale by two reference images.

In the image retrieval periods, two most similar images $I_{1},\;I_{2}$ are output. With camera calibrated, the scale can be computed from two images transformation and given pose. 

By applying epipolar constraint to image $I_{1},\;I_{2}$, we can get the transform matrix $T_{12}$ from $I_{1}$ to $I_{2}$:

(12) $$T_{12}=\left[\begin{array}{cc} R_{12} & t_{12}\\ 0 & 1 \end{array}\right],$$

Images $I_{1},\;I_{2}$ can be written in the form of homogeneous coordinate. Take image $I_{1}$ as example, the pose is $P_{1}=\left\{x_{1},\;y_{1},\;z_{1},\;qx_{1},\;qy_{1},\;qz_{1},\;qw_{1}\right\}$. Its translation is $t_{1}={\left[x_{1},y_{1},z_{1}\right]}^{T}$ and the rotation $R_{1}$ can be donated as Equation (13). Then image $I_{1}$ can be represented by transform matrix $T_{1}$ as Equation (14).

(13) $$R_{1}=\left[\begin{array}{ccc} 1-2qy_{1}{}^{2}-2qz_{1}{}^{2} & 2qx_{1}qy_{1}-2qw_{1}qz_{1} & 2qx_{1}qz_{1}+2qw_{1}qy_{1}\\ 2qx_{1}qy_{1}+2qw_{1}qz_{1} & 1-2qx_{1}{}^{2}-2qz_{1}{}^{2} & 2qy_{1}qz_{1}-2qw_{1}qx_{1}\\ 2qx_{1}qz_{1}-2qw_{1}qy_{1} & 2qy_{1}qz_{1}+2qw_{1}qx_{1} & 1-2qx_{1}{}^{2}-2qy_{1}{}^{2} \end{array}\right],$$

(14) $$T_{1}=\left[\begin{array}{cc} R_{1} & t_{1}\\ 1 & 1 \end{array}\right],$$

Transformation $T_{12}^{\prime }$ from image $I_{1}$ to image $I_{2}$ can be computed from a function as Equation (15), where $inv\left(T_{1}\right)$ is the inverse of matrix $T_{1}$. The relationship of transformation $T_{12}^{\prime }$ and its corresponding rotation $R_{12}^{\prime }$ and translation $t_{12}^{\prime }$ is in Equation (16).

(15) $$T_{12}^{\prime }=inv\left(T_{1}\right)T_{2},$$

(16) $$T_{12}^{\prime }=\left[\begin{array}{cc} R_{12}^{\prime } & t_{12}^{\prime }\\ 0 & 1 \end{array}\right],$$

Comparing $T_{12}^{\prime }$ with transform matrix $T_{12}$ computed from 2D-to-2D feature correspondence, we find the rotation $R_{12}$ almost equals to $R_{12}^{\prime }$, however $t_{12}$ and $t_{12}^{\prime }$ show a great difference and contain a scale as Equation (17), where s is the scale.

(17) $$t_{12}^{\prime }=s\;t_{12},$$

#### 3.4.4. Pose Estimation of the Query Image

In Section 3.4.2, computation of transform matrix from two images is illustrated, and Section 3.4.3 gave the method of computing scale from two retrieved images. As the poses of retrieved images are known, the transform matrix can be calculated by one of the retrieved images. In our method, we use two of the most similar images $I_{1}$ and $I_{2}$ to determine the scale *s*, then estimate the query image’s pose $P_{0}$ from the transform matrix $T_{10}$ which is computed from the most similar image $I_{1}$ and the query image $I_{0}$, and the relationship between the pose $P_{0}$ of image $I_{0}$ and corresponding transform matrix from $T_{0}$ is illustrated in Equation (18). $P_{0}$ can be denoted as $P_{0}=\left\{x_{0},\;y_{0},\;z_{0},\;qx_{0},\;qy_{0},\;qz_{0},\;qw_{0}\right\}$.

(18) $$T_{0}=|\begin{array}{cccc} 1-2qy_{0}{}^{2}-2qz_{0}{}^{2} & 2qx_{0}qy_{0}-2qw_{0}qz_{0} & 2qx_{0}qz_{0}+2qw_{0}qy_{0} & x_{0}\\ 2qx_{0}qy_{0}+2qw_{0}qz_{0} & 1-2qx_{0}{}^{2}-2qz_{0}{}^{2} & 2qy_{0}qz_{0}-2qw_{0}qx_{0} & y_{0}\\ 2qx_{0}qz_{0}+2qw_{0}qy_{0} & 2qy_{0}qz_{0}+2qw_{0}qx_{0} & 1-2qx_{0}{}^{2}-2qy_{0}{}^{2} & z_{0}\\ 0 & 0 & 0 & 1 \end{array}|,$$

## 4. Experimental Evaluation

### 4.1. Data Acquisition

For our experiment setup, we utilize images and their poses from trajectories for visual odometer since the task of visual positioning is similar to visual odometer tasks. In this experiment, the ICL-NUIM dataset [60] and the TUM RGB-D dataset [61] are adopted. 

**Figure 6:**
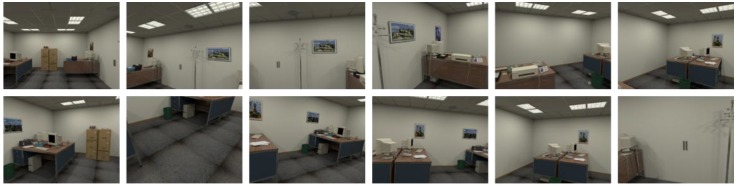
Images in office room scene of ICL-NUIM dataset.

**Figure 7:**
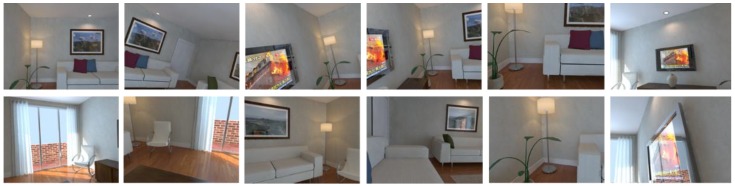
Images in living room scene of ICL-NUIM dataset.

**ICL-NUIM**: A dataset consists of RGB-D images from camera trajectories from two indoor scenes, *living room* and *office room*. The images were collected by a handheld Kinect RGB-D camera and the ground truth of trajectories was obtained by using Kintinuous [62]. The images were captured at 640 × 480 resolutions. Four trajectories were recorded in each scene, and images were taken at different positions for different trajectories. Images obtained at different pose are shown in Figure 6 and Figure 7.

**TUM RGB-D**: A dataset contains the color and depth images of a Microsoft Kinect sensor and the ground-truth trajectory of camera pose with the goal of establishing a benchmark for the evaluation of visual SLAM systems. The images are at a resolution of 640 × 480 and ground-truth trajectory was obtained from high accuracy motion-caption system. The dataset consists of 89 sequences from different camera motions.

**Figure 8:**
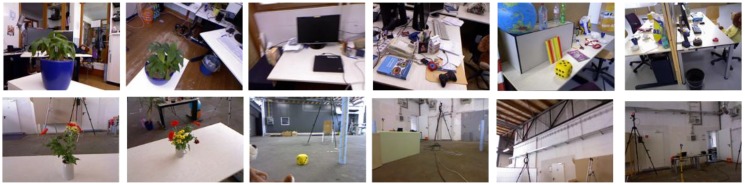
Images in TUM RGB-D dataset.

These datasets are employed to verify the performance of the proposed method, and images deal with different scales of captured area vary in capabilities of representing the scenario in different levels. Among these datasets, as shown in Figure 6, images in office scene of ICL-NUIM can represent a larger area such as half a room, whereas as shown in Figure 8, area represented in TUM RGB-D varies from a corner to part of room. As illustrated in Table 2, we choose a part of images in the dataset to represent scenarios, the number of train images and test images are also shown in this table. It should be noted that, database images are hand-picked to cover the application scenarios. The intrinsic parameters of the RGB camera can be obtained from Reference [63].

### 4.2. Performance of Image Retrieval

In this section, we evaluate the image retrieval performance by means of feature extraction methods on our database. It is important to note that, the process of evaluating the performance of image retrieval is time-consuming since feature extraction period is expensive, nevertheless, this process is not needed by our applying visual positioning algorithm.

Generally, mean average precision (mean AP) is applied to evaluate the performance of image retrieval task quantitatively, which compares the query image and the top retrieved images belonging to the same categories. The comparison of traditional image retrieval methods with CNN-based image retrieval methods has illustrated in Reference [29,64]. However, in the procedure of proposed image retrieval based visual positioning, mean AP cannot effectively demonstrate the retrieval result efficiently. Due to the feature extraction and matching period can affect the result of pose estimation heavily, pairs of images should share as many feature points as possible, which makes it essential for the query image and the retrieved images share some common areas. Therefore, we calculate the number of matched features between images to evaluate the result of image retrieval. 

To evaluate the performance of image retrieval, we extract feature points and descriptors from each pair of images, and calculate the number of good-match. In our retrieval period, three of the most similar images are returned. We extract ORB features from these retrieved images together with the query image, then match features of the query image and its corresponding retrieved images. Noted that Hamming Distance is employed to compute the distance between ORB descriptors. Then the minimal distance of matched descriptors is computed in all image pairs, and matched feature points whose distance is less than a threshold value can be labeled as good-match. Moreover, when calculating the good-match in ORB descriptors, the threshold value is defined by the larger number between twice of the minimal distance and a constant, since sometimes the minimal Hamming distance can be quite small. As shown in Table 3, a great number of good-matches are detected, which is sufficient for eight-point-algorithm in pose estimation period. The experiment results show that top-ranked similar images share more good-match.

In Reference [65], Jason et al. also developed an image based indoor localization scheme which uses FLANN search on SIFT features, and their experiment only successfully matched 78 out of 83 images to achieve a 94% retrieval accuracy. Whereas, in our proposed method, which aided by CNN-based image retrieval, achieved more than 99% image retrieval rate of 8267 images (the output images share the common area with the query image) owing to CNN features have more powerful representations for images.

### 4.3. Localization Results and Analysis

**Figure 9:**
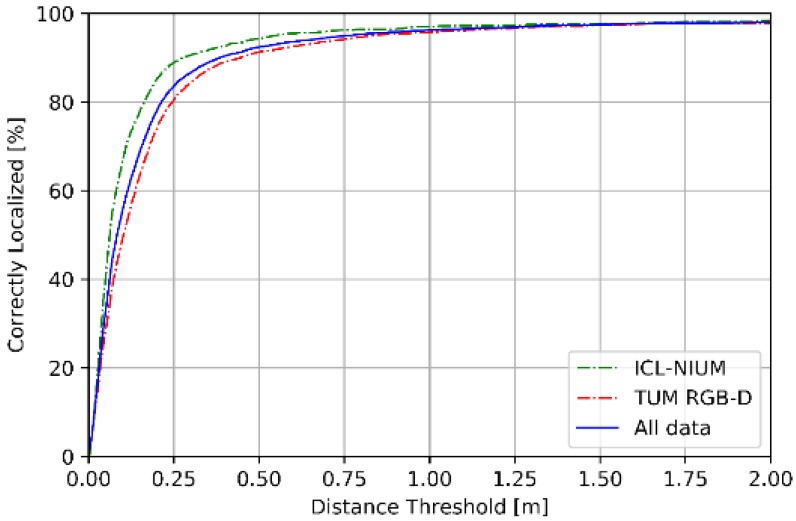
Cumulative distribution function of location error.

**Figure 10:**
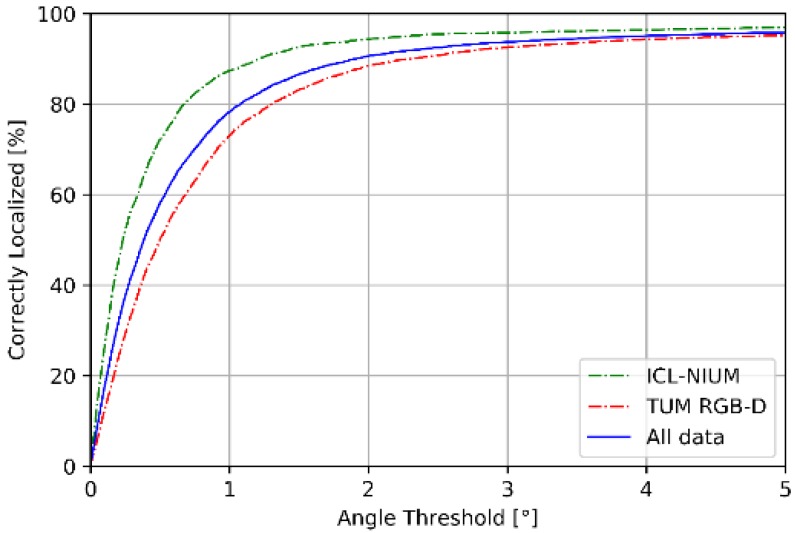
Cumulative distribution function of angle error.

Figure 9 and Figure 10 summarize the performance of pose estimation stage of proposed scheme. As shown in Figure 9a, our method is able to localize the position within sub-meter level of accuracy for over 90% of the query images in both datasets. Furthermore, more than 80% of the query images are successfully localized within 0.25 m of the ground truth position. As shown in Figure 9b, about 90% of the query images are localized within 3 degrees of ground truth position. We reported the performance in terms of the median errors of translation and orientation for each scene in the datasets, as shown in Table 4. The median errors of translation of our proposed method are around sub-meter level, which the 90% accuracy is 0.28 m in ICL-NUIM dataset and 0.45 m in TUM RGB-D dataset. The median error of orientation is within 1° and the 90% accuracy is 0.94° for ICL-NUIM dataset and 2.03° for TUM RGB-D dataset. It is important to note that the statistics in Table 4 has not removed the outliers, which enlarged the mean error of localization.

The proposed localization method combines CNN features and point features to estimate the pose. We compare the accuracy of the proposed method with the average pose estimation errors of three different CNN-based localization methods: (i) PoseNet which directly regress the camera pose by CNN; (ii) 4D PoseNet which was modified from PoseNet to accommodate the RGB-D input; (iii) CNN+LSTM [42], which utilize the PoseNet as a baseline pose estimator and the LSTM works as a temporal filter to process the estimated pose sequence. Table 5 summarizes statistics of the average pose estimation errors from those methods on ICL-NUIM dataset. We achieved better position accuracy on both scenarios, and achieved comparable accuracy in orientation.

More importantly, compared with state-of-the-art learning-based methods, the proposed scheme uses much fewer images in database construction period, which is significant for generalizing application of visual positioning. As is known, learning-based methods need quantity of images with poses to train a model (see Table 6). However, in proposed visual localization scheme, much fewer images are required. Less than 10% images are needed compared to learning-based methods, and we can still achieve comparable localization accuracy. Furthermore, CNN features can reduce the cost of model storage. Our CNN features model takes up 1.8 M storage for 886 images from TUM RGB-D database, which raw images occupy memory of 419.4 M, and 1.2 M storage is needed for 593 images from ICL NUIM database, which raw RGB images need 175.1 M.

We implemented the proposed localization scheme on Intel Core i7-7700 CPU @ 3.60 GHz. It takes 324.5 ms on average to find 10 best matches for a single image on 593 images of ICL NUIM database, and 336.5 ms to find 10 best matches on 886 images of TUM RGB-D database. Pose estimation costs 88.5 ms on average. The whole procedure from image retrieval to pose estimation takes ~0.45 s to output a location for a single image. We also employed a NVIDIA TITAN XP GPU to accelerate the computation of image retrieval, and 20 ms and 12 ms are taken to find 10 best matches of ICL NUIM and TUM RGB-D respectively. The whole procedure takes ~0.1 s for a single image. Computation and storing the CNN presentation of database images are done offline, and the period of retrieval evaluation, which is illustrated in Section 4.2, is unnecessary in localization procedure.

## 5. Discussion

In this study, we presented an image retrieval aided approach for indoor visual localization. A CNN-based image retrieval method was adopted to recognize the given query images by retrieving the matching images that were geo-tagged. The CNN-based strategy not only provides the output images with high spatial correlation, but also gives a new idea of scene representation. To put it another way, we no longer have to represent the space by its 3D model, instead we can design its spatial model representing methods in line with the usage of the spatial model. For instance, a group of CNN features with original images and poses can represent the whole area for visual localization purpose. A feature-points-correspondences strategy was then applied to estimate the precise location and pose of the query image. Experimental results demonstrated that our *monocular visual odometer*-inspired pose estimation methods resulted in high-precision localization consequence. 

It is obvious that our result outperformed that of retrieval-based methods without CNN feature extraction in robustness, due to the complex and unstable indoor environments. Compared with 2D-to-3D and 3D-to-3D methods in pose estimation methods, our strategy only depends on calibrated monocular camera in online localization period. Furthermore, our method is free of building 3D models, which the process is considered as an expensive process. Compared with end-to-end learning-based methods that directly regress the pose from input images, the advantage of our method exists in offline preparing period. As is known, deep learning has become an extremely powerful tool in computer vision tasks, but due to deep learning is a kind of data-hungry method, a massive of high-quality training data is required. Experimental results have shown that when achieving comparable localization accuracy, the number of database images of our method is far smaller than that in learning-based methods. Besides, learning-based strategies heavily rely on the training stage with quantity of geo-tagged images, it is assumed that when expanding the applicant area will cause the whole model retrained as well as the growth of model size, whereas in our methods, the increasing of raw data has no effect on the extractor, but only correspondingly add to the database. 

Moreover, owing to the scheme of data acquisition and the process of image-based localization, our method has massive potential to extend to a crowdsourcing-based method. The raw data from different resources can be integrated to compose the database. In the indoor environment, images are captured by cameras on cellphones, robots or other platforms, and the pose information can be obtained through pose measuring infrastructures. In fact, our proposed scheme is not a data-hungry solution as DCNNs, a set of limited images with high-precision pose is the key. In the future, the problems we need to address are the strategy of defining the space by a set of images and the approach to get high-precision pose information for database images. For image retrieval phase, a more efficient and robust method as well as more complicated and larger scale of environment needs to be considered in the future work.

## 6. Conclusions

In summary, our solution is highly available to different and complex environments and easily extendable to the change of raw data. We utilize a CNN-based image retrieval strategy which represents the scene by CNN features, and match the query image with database images. After that, the pose of the query image is recovered from the ORB feature points’ correspondence, which is efficient and effective. 

Based on the state-of-the-art studies of indoor visual localization systems, to the best of our knowledge, this work is the first to adopt both CNN-based image retrieval strategy and merely RGB images for accurate localization which is highly applicable to monocular vision positioning task. We think the image-based localization methods may become the mainstream owing to the scheme of data acquisition and the algorithm of pose estimation accorded with the current state of data expansion. The from-coarse-to-accurate strategy will be efficiently adopted to much larger applied range.

## Figures and Tables

**Table 1:** Composition of database.

Scene Labels	Color Images	Pose Information	CNN Features
$S_{1}$	$\left\{I_{11},I_{12},\;\ldots ,\;I_{1k_{1}}\right\}$	$\left\{P_{11},P_{12},\;\ldots ,\;P_{1k_{1}}\right\}$	$\left\{C_{11},C_{12},\;\ldots ,\;C_{1k_{1}}\right\}$
$S_{2}$	$\left\{I_{21},I_{22},\;\ldots ,\;I_{2k_{2}}\right\}$	$\left\{P_{21},P_{22},\;\ldots ,\;P_{2k_{2}}\right\}$	$\left\{C_{21},C_{22},\;\ldots ,\;C_{2k_{1}}\right\}$
…	…		
$S_{n}$	$\left\{I_{n1},I_{n2},\;\ldots ,\;I_{nk_{n}}\right\}$	$\left\{P_{n1},P_{n2},\;\ldots ,\;P_{nk_{n}}\right\}$	$\left\{C_{n1},C_{n2},\;\ldots ,\;C_{nk_{1}}\right\}$

**Table 2:** We chose images from different scenarios of ICL-NUIM and TUM RGB-D to compose our own experiment dataset.

Dataset	Scenario	The Number of Raw Images	The Number of Database Images	The Number of Test Images
ICL-NUIM	office room	4602	289	1495
	living room	4602	304	1533
TUM RGB-D	freiburg1_plant	1141	115	456
	freiburg1_room	1362	91	454
	freiburg2_360_hemisphere	2729	273	1092
	freiburg2_flowerbouquet	2972	149	1188
	freiburg2_pioneer_slam3	2544	128	1017
	freiburg3_long_office_household	2585	130	1034

**Table 3:** The average number of ORB Oriented FAST and Rotated BRIEF) good-match in two datasets.

Similarity Rank	ICL-NUIM Dataset (Test on 3026 Images)	TUM RGB-D Dataset (Test on 5241 Images)
1	252.8	225.3
2	203.1	135.9
3	158.4	104.0
Average	204.8	155.1

**Table 4:** Localization performance in different scenarios from different datasets.

Dataset	Scenario	The Median Error	The Mean Error	90% Accuracy
ICL-NUIM	office room	0.07 m 0.01°	0.31 m 2.47°	0.35 m 0.83°
	living room	0.05 m 0.02°	0.36 m 4.36°	0.23 m 1.03°
	**All images**	0.06 m 0.01°	0.34 m 3.43°	0.28 m 0.94°
TUM RGB-D	freiburg1_plant	0.12 m 0.01°	0.38 m 3.37°	0.45 m 1.95°
	freiburg1_room	0.17 m 0.54°	0.43 m 4.82°	0.71 m 4.04°
	freiburg2_360_hemisphere	0.05 m 0.16°	0.38 m 6.55°	0.38 m 1.08°
	freiburg2_flowerbouquet	0.07 m 0.12°	0.15 m 5.32°	0.26 m 2.54°
	freiburg2_pioneer_slam3	0.13 m 0.13°	0.34 m 8.80°	0.66 m 1.54°
	freiburg3_long_office_household	0.15 m 0.21°	0.36 m 3.00°	0.41 m 2.05°
	**All images**	0.10 m 0.16°	0.32 m 5.58°	0.45 m 2.03°

**Table 5:** Comparison of average pose estimation error in ICL-NUIM dataset.

Method	Living Room	Office Room
PoseNet	0.60 m, 3.64°	0.46 m, 2.97°
4D PoseNet	0.58 m, 3.40°	0.44 m, 2.81°
CNN+LSTM [42]	0.54 m, 3.21°	0.41 m, 2.66°
**ours**	**0.36 m**, 4.36°	**0.31 m**, **2.47°**

**Table 6:** Comparison of database sizes.

Method	Database Image per Scene	Median Localization Error
PoseNet	3000	0.47 m 14.40°
	6000	0.48 m 7.68°
NNnet [66]	2000	0.27 m 11.82°
	4000	0.24 m 6.35°
VLocNet	2000	0.097 m 6.48°
	4000	0.036 m 1.71°
**ours**	148	0.102 m 0.164°
	289	0.062 m 0.011°
